# Anti-schistosomal Intervention Targets Identified by Lifecycle Transcriptomic Analyses

**DOI:** 10.1371/journal.pntd.0000543

**Published:** 2009-11-03

**Authors:** Jennifer M. Fitzpatrick, Emily Peak, Samirah Perally, Iain W. Chalmers, John Barrett, Timothy P. Yoshino, Alasdair C. Ivens, Karl F. Hoffmann

**Affiliations:** 1 Department of Pathology, University of Cambridge, Cambridge, United Kingdom; 2 Institute of Biological, Environmental and Rural Sciences (IBERS), Aberystwyth University, Aberystwyth, United Kingdom; 3 Department of Pathobiological Sciences, School of Veterinary Medicine, University of Wisconsin, Madison, Wisconsin, United States of America; 4 Fios Genomics, ETTC, King's Buildings, Edinburgh, United Kingdom; McGill University, Canada

## Abstract

**Background:**

Novel methods to identify anthelmintic drug and vaccine targets are urgently needed, especially for those parasite species currently being controlled by singular, often limited strategies. A clearer understanding of the transcriptional components underpinning helminth development will enable identification of exploitable molecules essential for successful parasite/host interactions. Towards this end, we present a combinatorial, bioinformatics-led approach, employing both statistical and network analyses of transcriptomic data, for identifying new immunoprophylactic and therapeutic lead targets to combat schistosomiasis.

**Methodology/Principal Findings:**

Utilisation of a Schistosoma mansoni oligonucleotide DNA microarray consisting of 37,632 elements enabled gene expression profiling from 15 distinct parasite lifecycle stages, spanning three unique ecological niches. Statistical approaches of data analysis revealed differential expression of 973 gene products that minimally describe the three major characteristics of schistosome development: asexual processes within intermediate snail hosts, sexual maturation within definitive vertebrate hosts and sexual dimorphism amongst adult male and female worms. Furthermore, we identified a group of 338 constitutively expressed schistosome gene products (including 41 transcripts sharing no sequence similarity outside the Platyhelminthes), which are likely to be essential for schistosome lifecycle progression. While highly informative, statistics-led bioinformatics mining of the transcriptional dataset has limitations, including the inability to identify higher order relationships between differentially expressed transcripts and lifecycle stages. Network analysis, coupled to Gene Ontology enrichment investigations, facilitated a re-examination of the dataset and identified 387 clusters (containing 12,132 gene products) displaying novel examples of developmentally regulated classes (including 294 schistosomula and/or adult transcripts with no known sequence similarity outside the Platyhelminthes), which were undetectable by the statistical comparisons.

**Conclusions/Significance:**

Collectively, statistical and network-based exploratory analyses of transcriptomic datasets have led to a thorough characterisation of schistosome development. Information obtained from these experiments highlighted key transcriptional programs associated with lifecycle progression and identified numerous anti-schistosomal candidate molecules including G-protein coupled receptors, tetraspanins, Dyp-type peroxidases, fucosyltransferases, leishmanolysins and the netrin/netrin receptor complex.

## Author Summary

Despite the implementation of focused and well-funded chemotherapeutic control initiatives over the last decade, schistosomiasis remains a significant cause of morbidity and mortality within countries of the developing world. There is, therefore, an urgent need for the rapid translation of genomic information into viable vaccines or new drug classes capable of eradicating the parasitic schistosome worms responsible for this neglected tropical disease. In our effort to identify potential targets for novel chemotherapeutic and immunoprophylactic interventions, we detail a combined bioinformatics approach, comprising exploratory statistical and network analyses, to thoroughly describe the transcriptional progression of *Schistosoma mansoni* across three environmental niches. Our results indicate that, although schistosomes are masters at host deception and survival, there are numerous exploitable candidate molecules displaying either differential or constitutive expression throughout the parasite's lifecycle. Importantly, some of these transcripts represent gene families not commonly found outside—or expanded within—the phylum Platyhelminthes, and thus represent priority targets. Many of the candidates identified herein will be subjected to ongoing and future hypothesis-led functional investigations. The completion of such specific examinations ultimately will contribute to the successful development of novel control strategies useful in the alleviation of schistosome-induced immunopathologies, morbidities and mortalities.

## Introduction

Parasitic helminths constitute the most important group of metazoan pathogens affecting the global health and wellbeing of human and animal populations. While most infected individuals live within subtropical and tropical countries of the developing world, distribution of favourable habitats for helminth transmission will likely expand considering the prediction of a worldwide temperature increase between 1.1°C–6.4°C in the 21^st^ century [1]. Worryingly, existing control strategies are extremely restricted; the main weapon being limited anthelmintics to treat infected individuals therapeutically. Additionally, there are no commercial vaccines available to combat infection prophylactically and the general community consensus is that no current experimental vaccine will be available for widespread use in the foreseeable future [2].

Despite these disturbing trends and problematic issues, recent genomic and transcriptomic characterisation of several nematode and platyhelminth species have provided the necessary resources for post-genomic technologies to make positive strides in the identification of novel anthelmintic targets [3],[4],[5],[6]. In the research field of schistosomiasis, a disease that kills 200,000 individuals/annum [7], elucidation of the expressed genome complement [4],[5], assembly and annotation of first pass genomes [8],[9], multiple descriptions of targeted proteome, glycome and differential transcriptomes [10],[11] and the utilisation of post-transcriptional gene silencing (PTGS) [12] have all contributed to a much greater understanding of the causative parasite's biology. Collectively, this information has vastly accelerated our ability to identify and prioritise next generation drug and vaccine targets.

A potential bottleneck in exploiting this knowledge for schistosomiasis control, however, relates to the complexity of the schistosome's digenetic and dioecious lifecycle. As the parasite alternates between three environmental niches (freshwater, intermediate molluscan and definitive vertebrate hosts) and undergoes two distinct types of reproductive processes (asexual and sexual), it is experimentally difficult to identify when and where all of the 13,000 gene products are expressed [8],[9]. However, categorisation of the expressed genome complement during schistosome development has recently been conducted in a few high-throughput DNA microarray studies [13],[14],[15]. While generally informative, these studies were, nevertheless, limited by insufficient surveying of lifecycle stages, use of restricted DNA microarrays or utilisation of traditional statistical analyses not adjusted for false discovery rates. Improvements in the methodology used for analysing DNA microarray data as well as profiling larger numbers of schistosome life-stages will ultimately provide a more detailed interpretation of parasite developmental biology useful for target identification. Towards this end, we describe the combined use of a 37,632 element long-oligonucleotide DNA microarray [16], pairwise statistical comparisons and network theory analyses [17] to thoroughly characterise the progression of schistosome development spanning intra-molluscan- (mother sporocyst, daughter sporocyst), aquatic-dwelling- (egg, cercariae and miracidia) and intra-vertebrate- (egg, 3-hr, 24-hr, 3-day, 6-day, 2-wk, 3-wk, 5-wk, 7-wk, adult males and adult females) lifecycle stages.

It is clear from this study that combined statistical and network-based data analyses provide greater characterisation of schistosome developmental processes than employing either single approach alone. Using this information, we thoroughly describe the transcriptional profiles associated with the dioecious and digenetic state, detail multiple subsets of stage-specific transcripts, identify a cohort of constitutively expressed gene products and report that the egg and daughter sporocyst life-stages display highly divergent transcriptional profiles. Furthermore, we demonstrate the added value in simultaneously comparing the developmental expression profiles of selected gene family members described as the next generation vaccine candidates (the tetraspanins [18]), drug targets (G-protein coupled receptors [19]) or enzymes involved in producing immunomodulatory epitopes (fucosyltransferases [20]). Finally, we illustrate how global gene expression profiles can provide vital developmental information related to transcripts that have no known sequence similarity outside of the phylum Platyhelminthes, and thus, may represent new classes of anthelmintic targets.

## Methods

### Parasite material

A Puerto Rican strain of *S. mansoni* was used in this study. All procedures performed on mice adhered to the United Kingdom Home Office Animals (Scientific Procedures) Act of 1986 and were approved by Aberystwyth University's ethical review panel (ERP). Mixed-sex worms (including gender-separated, mature adult male and female worms) were perfused from percutaneously infected TO (Tuck Ordinary) mice (Harlan, UK) challenged 2-, 3-, 5- or 7-weeks earlier with 250 cercariae [21]. Eggs were recovered from mouse livers of animals infected 7-weeks prior with cercariae as previously described [21]. Cercariae were shed from *Biomphalaria glabrata* intermediate snail hosts. Schistosomula were prepared by mechanical transformation [21] and cultured at 37°C in DMEM (Sigma, UK) supplemented with 10% foetal calf serum, 2 mM *L*-glutamine, and 100 µg/ml penicillin/streptomycin in an atmosphere of 5% CO_2_ for the timeframe indicated (3 hr, 24 hr, 3-day, 6-day). Miracidia were hatched from *S. mansoni* eggs harvested from TO mouse livers seven weeks after infection. Mother (2-day) sporocysts were obtained by *in vitro* transformation of isolated miracidia [22], while daughter sporocysts were derived from 15-day infected *B. glabrata* snails [23].

### Genomic DNA isolation

*S. mansoni* genomic DNA (gDNA) was prepared from mixed-sex cercariae using a commercially available kit (DNeasy Tissue Kit, Qiagen UK). All gDNA was qualitatively assessed by agarose gel electrophoresis and quantitatively measured using a NanoDrop ND-1000 UV-Vis spectrophotometer.

### Total RNA isolation

*S. mansoni* total RNA was isolated as previously described [24]. Isolated total RNA was treated with DNAse I (Ambion UK) to ensure RNA free from gDNA contamination. All RNA was subsequently quantified with a NanoDrop ND-1000 UV-Vis spectrophotometer and visualized for quality using an Agilent 2100 bioanalyzer.

### Construction of long oligonucleotide DNA microarray

The *S. mansoni* long oligonucleotide DNA microarray was designed and constructed as reported in Fitzpatrick *et al*. [16]. Briefly, the DNA microarray consists of 37,632 elements; 35,437 *S. mansoni* oligonucleotide 50-mers and 2,195 controls. The microarray is deposited in the ArrayExpress (European Bioinformatics Institute, Hinxton UK) database under the accession number A-MEXP-830 and is available from the Centre for Microarray Resources, University of Cambridge UK. Sequences of each oligonucleotide 50-mer and positions of stringent alignment on the *S. mansoni* genome can be visualized using the *S. mansoni* genome browser at GeneDB [25]. All clustered parent sequences used for oligonucleotide design can be found in Dataset S1.

### DNA microarray hybridization

AlexaFluor647-dCTP (AF647 - Amersham Biosciences UK) labeled cDNA targets were generated through a modified version of the procedure first described by Petalidis *et al.* [26]. These cDNA targets were co-hybridised to the *S. mansoni* long-oligonucleotide DNA microarray with a reference gDNA sample, labeled with AlexaFluor555-dCTP (AF555) as previously described [16]. Image acquisition (16-bit tiff) for the DNA microarray was performed using a GenePix® 4100A (Axon Instruments Inc.) dual channel laser scanner at 10 µm resolution, 100% laser power and photomultiplier tube gain levels ranging from 730 to 870. BlueFuse for Microarrays (BlueGnome Ltd., UK) image analysis software was employed to extract fluorescent signal intensity data.

### DNA microarray data analysis

‘Universal Timepoint Codes’ were assigned to the lifecycle stages as follows: egg UT01, miracidia UT02, mother (2-day) sporocysts UT03, daughter sporocysts UT04, cercariae UT05, 3-hr somules UT06, 24-hr somules UT07, 3-day somules UT08, 6-day somules UT09, 2-wk worms UT10, 3-wk worms UT11, 5-wk worms UT12, mixed-sex 7-wk worms UT13, male 7-wk worms UT14, female 7-wk worms UT15.

Array data, normalised within each microarray to correct for local background (print tip loess normalisation), were subsequently normalised across all arrays using quantile normalisation with the reference channel (gDNA; AF555). The log_2_ values of the AF647 channel data (non-control features only) were then extracted for further analysis. All microarray data are MIAME compliant [27] and a full (including controls) set of raw and normalised data is available via ArrayExpress [28] under the experiment accession number E-MEXP-2094.

### Statistical analysis of DNA microarray data

Normalised log_2_ AF647 intensities for all DNA microarray elements (minus negative controls) available for all 15 lifecycle stages were linear model fitted using 3 replicates/lifecycle stage (except for the adult female lifecycle stage where one replicate was manually excluded). The Student's *t*-test incorporating a Benjamini & Hochberg *p*-value adjustment (for multiple testing; the adjusted *p*-value controls for false discovery rate) was used to identify statistically significant differences in gene expression between any compared life-stage. Log odds (B values) were also calculated; transcripts having B values >0 are more likely to be differentially expressed than not differentially expressed in any pairwise comparison (full list of comparisons included in Table 1, Dataset S2 and Dataset S3).

**Table 1:** Pairwise comparisons of key schistosome developmental stages reveal a number of differentially expressed genes.

	Numbers of differentially expressed transcripts identified between indicated pair wise comparison^a^						
Life-stage comparison	*p*<0.05	*p*<0.01	*p*<0.001	*p*<0.0001	Adj*p*<0.05**^b^**	Adj*p*<0.01**^b^**	B≥0**^c^**
Miracidium/Egg	3990	1677	444	67	48	0	219
Mother Sporocyst/Miracidium	147	48	9	4	1	0	0
Daughter Sporocyst/Mother Sporocyst	1971	378	53	14	4	2	11
Cercariae/Daughter Sporocyst	2353	680	202	87	103	47	141
Cercariae/Miracidium	860	462	223	137	166	98	186
3-Hr Schistosomulum/Cercariae	159	30	4	0	0	0	0
24-Hr Schistosomulum/Cercariae	321	111	27	5	0	0	0
3-Day Schistosomulum/Cercariae	308	120	48	14	2	0	0
6-Day Schistosomulum/Cercariae	457	179	74	29	17	6	23
24-Hr Schistosomulum/3-Hr Schistosomulum	141	37	2	1	0	0	0
3-Day Schistosomulum/24-Hr Schistosomulum	40	14	4	1	0	0	0
6-Day Schistosomulum/3-Day Schistosomulum	65	9	2	0	0	0	0
2-Wk Worm/6-Day Schistosomulum	1728	411	62	18	8	2	23
3-Wk Worm/2-Wk Worm	3599	940	94	4	0	0	23
Intra-snail stages/Intra-mouse stages**^d^**	2608	1405	832	570	866	628	703
5-Wk Worm/3-Wk Worm	472	136	72	38	26	11	37
7-Wk Worm/5-Wk Worm	1119	219	17	3	0	0	0
7-Wk Worm/3-Wk Worm	274	134	82	47	41	16	52
7-Wk Female Worm/7-Wk Male Worm	609	209	85	43	31	5	58

(a) Pair wise comparisons represent the normalised log2 gene expression ratios for a gene in life stage ‘x’ relative to the same gene in sample ‘y’. (b) Adjusted *p* values (Adj*p*) are derived from Benjamini & Hochberg correction method (). (c) B represents natural log odds of *p* (transcript is differentially expressed)/*p* (transcript is not differentially expressed) and indicates those transcripts more likely to be differentially expressed than not (B≥0). (d) Intra-snail stages/Intra-mouse stage comparisons represent those genes that are differentially expressed as detected by the following formula: log2 gene expression ratios of (Miracidium + Daughter sporocyst + Mother Sporocyst + Cercariae)/4 compared to log2 gene expression ratios of (3-Hr schistosomules +24-Hr schistosomules +3-Day schistosomules +6-Day schistosomules +2-Wk worms +3-Wk worms +5-Wk worms +7-Wk worms +7-Wk Male worms +7-Wk Female worms/10). A top-table list containing these identified gene products is provided in Dataset S2.

Heat maps displaying expression data were generated by Empirical Bayesian analysis of the normalised AF647 values (Dataset S4). LogFC (fold change) was also calculated in some instances. Here, the logFC is the difference in log_2_ intensities for each of the array features between two different lifecycle stages (i.e. UT02-UT01 for gene X  =  log_2_intensity (UT02, gene X) – log_2_intensity (UT01, gene X)).

Those transcripts that exhibited the lowest 1% variation in log_2_ AF647 intensities across the entire lifecycle were identified as constitutively expressed gene products (Dataset S5).

### Network analysis

A data matrix of normalised, de-logged, intensity values for all genes across all microarrays was generated (Dataset S4). The Pearson correlation between every object (a single gene's normalised AF647 intensity values across all arrays) and every other object in that matrix was subsequently determined by Biolayout Express 3D [29], effectively calculating the similarity in the profile of expression of every gene on the DNA microarray across the samples analysed, to every other gene on the DNA microarray. All relationships above a Pearson correlation cut off of r = 0.94 were stored. The stored data were used to draw the edges (lines) between nodes (transcripts) in network graphs. Nodes sharing edges (relationships above the set threshold) form into groups (graph components). Connectivity between nodes determines the 3-dimensional layout of the graphs. Using a Pearson correlation cut off (r = 0.94), 16,621 nodes formed graph components.

Large graphs were further subdivided into groups or clusters of nodes using a Markov Cluster algorithm (MCL). MCL clusters included transcripts that were statistically differentially expressed across groups (i.e. Adj*p*-value <0.05) as well as those that, although exhibiting similar profiles, were not statistically significant. This type of analysis therefore allows for statistical associations to be verified, but also is capable of identifying associations which statistical analyses (on minimally replicated datasets) may have not been able to detect. MCL clustering applied to this dataset at a stringency of 1.7 was able to identify 387 clusters of 6 or more nodes (i.e. 12,132 nodes or 73% of the original dataset, Dataset S6).

### Gene Ontology (GO) analysis

A file containing all GO terms mapped to the *S. mansoni* gene models in GeneDB [25] was generated for analysis. To identify GO terms across all three categories (biological process, molecular function or cellular composition) that were enriched in the constitutively expressed gene list (Dataset S5), a hypergeometric test was performed. GO terms having a hypergeometric *p*-value <0.01 were considered enriched in the constitutive expressed gene products.

GO enrichment analysis of MCL cluster members was also performed, but was necessarily restricted to those with one or more Smp loci (as GO terms were mapped to Smp loci) and was not directional (i.e. could not be done separately for “up” and “down” regulated genes). As performed for the GO enrichment analysis of the constitutively expressed gene products, a hypergeometric enrichment *p*-value <0.01 was applied (Dataset S6).

### Gene family analysis

Three gene families were selected for detailed analysis. Nucleic acid sequences corresponding to the tetraspanins (PF00335), the fucosyltransferases (PF00852) and the G-protein coupled receptors (PF00001, PF00002 and PF00003) were downloaded from *Schistosoma mansoni* GeneDB [25], assembly version 4. Intra-family nucleic acid sequences were assembled into contigs and singletons according to the default parameters of SeqMan within the DNASTAR Lasergene application software. DNA microarray oligonucleotides mapping to each representative contig/singleton were identified (when possible) and normalised AF647 intensity units were extracted. Heatmaps were generated (Spearman's rank, average linkage clustering) for each gene family allowing gene expression values to be visualised for each member across the schistosome lifecycle.

### Real time reverse transcription (RT) - PCR analysis

The transcript abundance of each selected mRNA was quantified relative to alpha tubulin in eight stages (triplicate measurements) of the *S. mansoni* lifecycle using real-time RT-PCR analysis as previously described [30]. Reactions were performed on a MiniOpticon real-time PCR thermal cycler system (Bio-Rad) using iQ SYBR Green Master Mix (Bio-Rad) according to the manufacturer's instructions. The amplification efficiency (E) of each primer set was determined during assay development by plotting the cycle thresholds (C_t_) from serial dilutions of a suitable cDNA sample and inputting the resulting slope in the equation, E = 10^(−1/slope)^. For each real-time PCR reaction the following equation was used to calculate a normalised SmGeneX expression ratio: 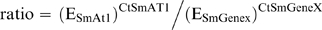
where E_SmAt1_ is the amplification efficiency of the reference gene (alpha tubulin), E_SmGeneX_ is the amplification efficiency of the target gene, CtGeneX is the cycle threshold of the target gene and CtSmAT1 is the cycle threshold of the reference gene from the same cDNA sample. PCR oligonucleotide primer specifics are included in Datasets S7–S9.

### BLAST facilitated genome exclusion analysis

Two FASTA files were generated for iterative BLAST analysis; one containing 612 sequences derived from 37 MCLs demonstrating transcription of genes in cercariae/schistosomula and/or definitive host lifecycle stages (Dataset S6) and one containing 338 (manual removal of 17 low quality sequences from the original 355) sequences derived from genes demonstrating less than 1% variability (Dataset S5) in expression across the schistosome lifecycle (constitutively-expressed). These two FASTA files were subsequently subjected to the same three iterative BLAST analyses. First, sequences were submitted to a BLASTn analysis against the *S. mansoni* shotgun reads collected from the Wellcome Trust Sanger Institute's genome project [31] to insure that they were indeed derived from schistosome material. Only those sequences displaying E values less than 1e^−10^ were retained. Secondly, the remaining sequences were submitted to a BLASTx search of the non-redundant protein databases to eliminate cross-genome orthologs (hits demonstrating E values less than 1e^−05^ were excluded). Finally, the remaining schistosome sequences were submitted to a tBLASTx search of the EST databases to further eliminate cross-genome hits contained within high-throughput sequencing projects (hits demonstrating E values less than 1e^−05^ were excluded). Gene products passing all three iterative BLAST criteria are included in Dataset S5 (constitutively-expressed transcripts) and Dataset S6 (developmentally-regulated transcripts).

## Results

### *Schistosoma* lifecycle correlation analyses

**Figure 1:**
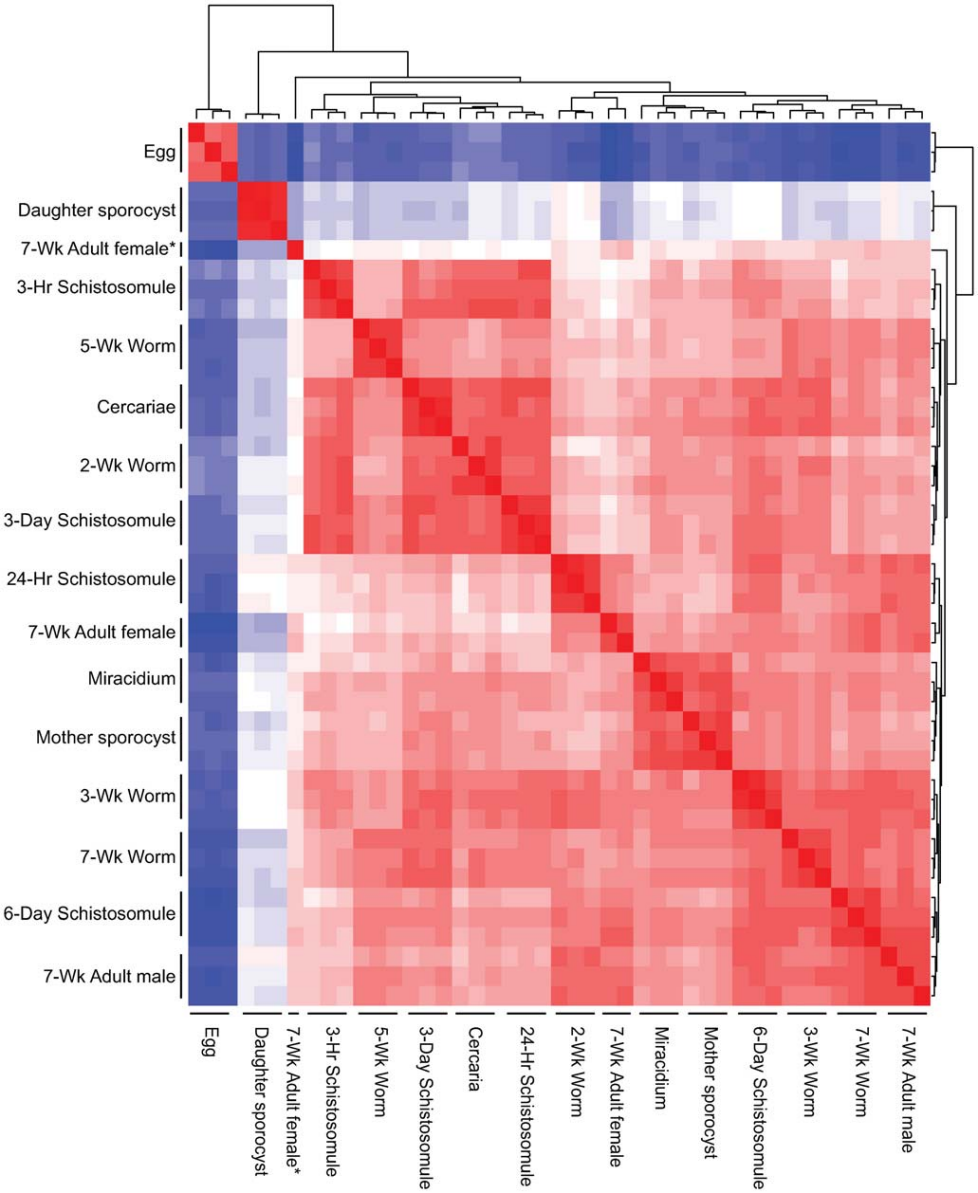
Egg and daughter sporocyst parasite stages display the most divergent transcriptional profiles within the *Schistosoma* lifecycle. Correlations of M (log_2_ (Alexafluor647/Alexafluor555)) ratio values between intra- (lifecycle replicate) and inter- (life-stage comparison) parasite samples were calculated. A heatmap of the correlations, calculated on data normalised within each DNA microarray, is illustrated. Red represents a high correlation and blue depicts a low or lower correlation. Un-scaled data were clustered by Euclidean distance and shown as dendrograms above and to the left of the heatmap. * Indicates female sample replicate manually removed.

This study details the transcriptional progression of schistosome development using DNA microarray profiling and combinatorial bioinformatics involving statistical (pairwise comparisons)- and network (graph clustering)- based data mining. Prior to bioinformatics-led data mining, rigorous normalisation of the lifecycle dataset was employed and M ratio (log_2_ (Alexafluor647/Alexafluor555 intensities)) correlations investigated to determine relationships between replicate samples and lifecycle stages (Fig. 1). Here, replicate expression values collected from DNA microarray hybridisation of the same lifecycle stage were highly correlated, with all but one adult female replicate demonstrating strong correlations with the other replicate samples. This adult female replicate was manually excluded from subsequent analyses. However, the most important observation from these M ratio correlation analyses was that, while most schistosome lifecycle stages were to some degree transcriptionally correlated, there were two striking exceptions. The egg (miracidium-producing) and the daughter sporocyst (cercariae-producing) life-stages were found to be outliers, demonstrating that each are transcriptionally distinct when compared to all other schistosome developmental forms.

### *Schistosoma* development characterised by pairwise statistical analyses

The first approach in describing the transcriptional patterns underlying schistosome development involved the identification of differential gene expression calculated from a series of pairwise comparisons between key lifecycle stages (Table 1 and Dataset S2). We chose an adjusted *p* value (Adj*p* <0.05), corrected using the Benjamini & Hochberg method for multiple testing [32], to identify differentially expressed genes between the paired comparisons. Using this criterion, nineteen different comparisons were performed with a total of 1,313 (973 non-redundant) gene products differentially expressed. Many of the differentially expressed transcripts confirmed patterns identical to those previously reported. For example, *S. mansoni* venom allergen-like molecules (SmVALs) 3 (CONTIG4696 and AI395850, Smp_193710) and 9 (CD111828, Smp_176180) were confirmed as differentially expressed in the snail-residing schistosome stages compared to mouse-residing parasite forms whereas SmVAL7 (SMLC15A11A, Smp_070240) and SmVAL13 (CONTIG2212, Smp_124060) displayed the opposite trend [30]. Other genes differentially expressed in this specific paired comparison (intra-snail vs. intra-mouse, Table 1) are likely to include transcripts associated with the biology of the digenetic state. Loosening of this strict statistical criterion (Adj*p* <0.05) identified additional gene products differentially expressed in all of the pairwise comparisons performed (Table 1 and Dataset S2). Many of these comparisons may be useful in staging (i.e. defining age) schistosome development.

**Figure 2:**
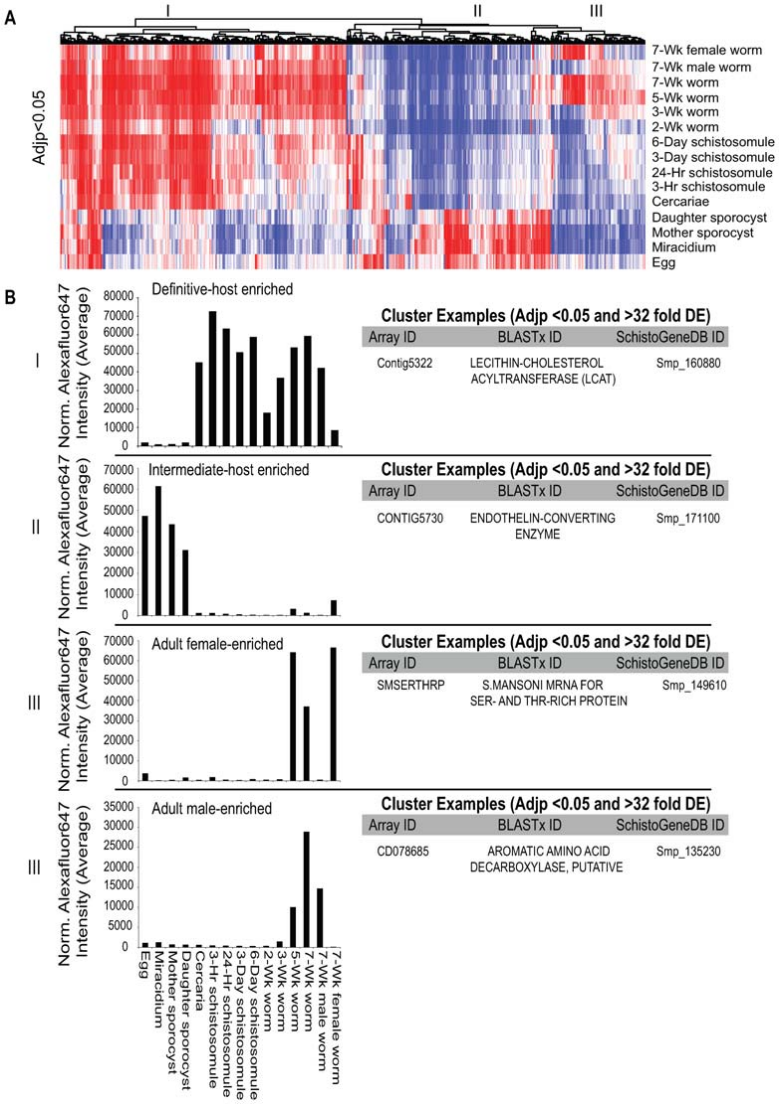
Clusters associated with the schistosome digenetic and dioecious states are the most statistically significant transcriptional patterns identified. Normalised and *p* value corrected data (see *Methods*) were used to generate statistically significant gene lists (Table 1, Datasets S2 & S3). A) A heatmap of differentially expressed genes significant at an adjusted *p* value of <0.05 in at least one bimodal life stage comparison is illustrated (red – highly expressed, blue – weakly expressed). Log_2_ intensity values were clustered by Euclidean distance and shown as a dendrogram on top of the heatmap. Three major clusters are identified (I – expressed predominantly in the definitive host, II – expressed predominantly in the intermediate host and III – expressed differentially between the adult sexes). B) Specific examples of some transcripts found in each of the three major clusters additionally showing >32-fold differential expression (DE) in at least one bimodal life-stage comparison. A full list of transcripts showing >32 fold DE in at least one bimodal life-stage comparison is found in Dataset S3. Bar graphs represent average normalised AF647 intensity values (Dataset S4) for the chosen transcripts. Array ID represents unique 50-mer oligonucleotide identifier on the DNA microarray (list of parent sequences where 50-mer oligonucleotide was designed is found in Dataset S1); BLASTx ID represents name of closest match to parent oligonucleotide sequence in NCBI database; SchistoGeneDB ID represents gene model (version 4.0) identifier of the mapped 50-mer oligonucleotide.

Collective visualisation of all 973 non-redundant differentially expressed gene products (Dataset S3) as a heatmap illustrated three major patterns of transcription: 1) genes expressed predominantly in the vertebrate dwelling schistosome life-stages (definitive host-enriched, Fig. 2A, clade I), 2) genes primarily expressed in the snail dwelling schistosome life-stages (intermediate host-enriched, Fig. 2A, clade II) and 3) genes mainly expressed in adult parasites (female- or male- enriched, Fig. 2A, clade III). This was not surprising as these patterns associate with the major biological features of the schistosome's lifecycle; digenesis and dioecy. Further filtering of this dataset to include only transcripts that additionally contain a greater than 32-fold differential expression (in at least one pairwise comparison) revealed the most developmentally regulated schistosome genes across the parasite's lifecycle (Fig. 2B and Dataset S3). These 448 transcripts included an aquaporin (aquaporin-3: Contig45, Smp_005720), a lecithin-cholesterol acyltransferase (LCAT: Contig5322, Smp_160880), an endothelin-converting enzyme (neprilysin-2: CONTIG5730, Smp_171100), a putative aromatic amino acid decarboxylase (CD078685, Smp_135230) and a *S. mansoni* mRNA encoding a Ser- and Thr- rich mucin-like protein (SMSERTHRP, Smp_149610) containing a C-type lectin domain. Interestingly, four well-studied gene families were also highly enriched within this developmentally-regulated dataset; the previously mentioned SmVALs (unknown function [30]), the tetraspanins (putative vaccine candidates [18]), the tegument-associated antigens (associated with resistance to re-infection in human populations [33]) and the cathepsin B/Ls (involved in proteolytic biology of schistosome haematophagy [34]).

**Figure 3:**
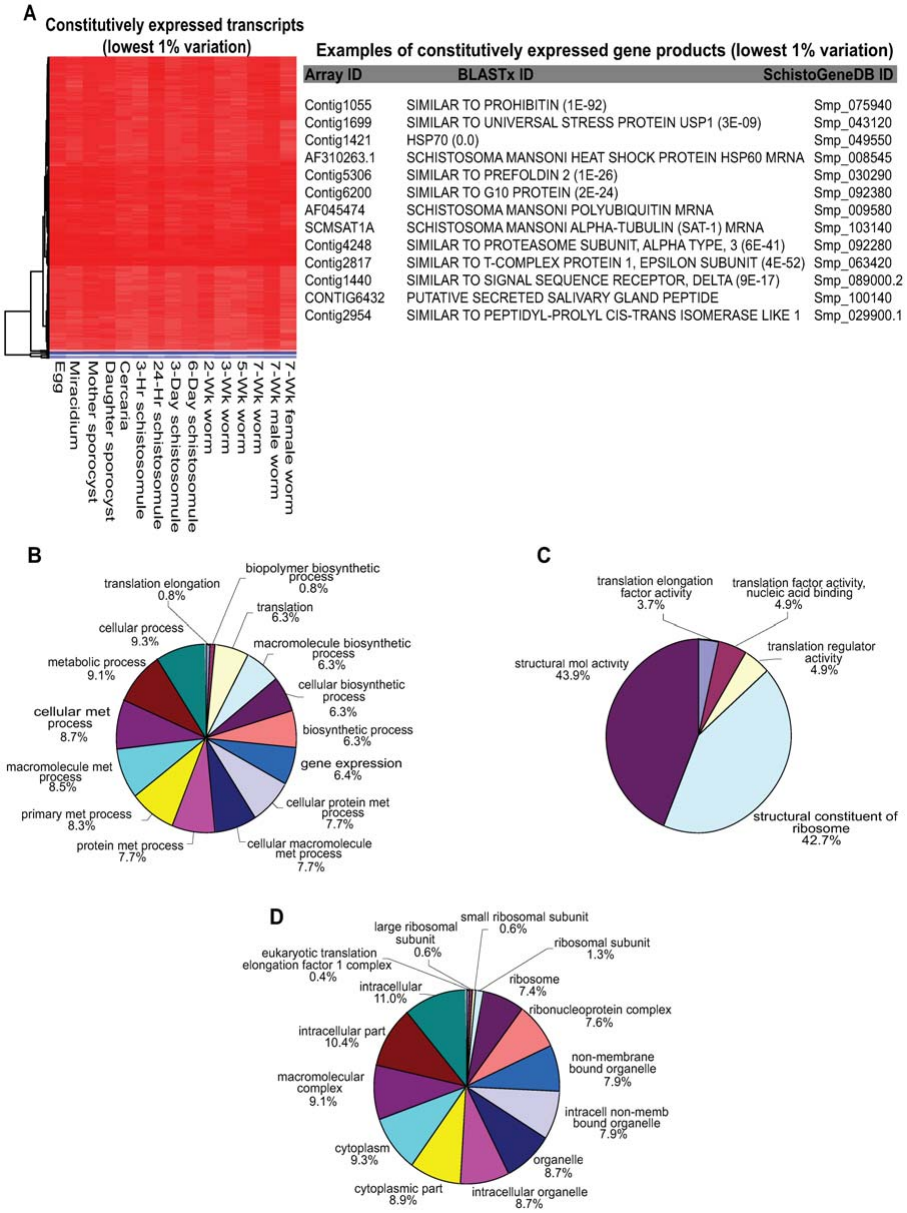
A cohort of constitutively expressed gene products is identified from DNA microarray analysis of the *Schistosoma* lifecycle. Normalised log_2_ AF647 intensity data were used to identify gene products constitutively expressed throughout the schistosome lifecycle (Dataset S5). A) Heatmap of transcripts exhibiting the lowest levels of variation (1% variation across lifecycle). Log_2_ intensity values were clustered by Euclidean distance and shown as a dendrogram to the left of the heatmap (red – highly expressed, blue – weakly expressed). Specific examples of gene products constitutively expressed are illustrated (full list included in Dataset S5). B–D) Oligonucleotide sequences (Array ID) mapping to specific *S. mansoni* gene models (version 4.0) in SchistoGeneDB were analysed for Gene Ontology enrichment (*p*<0.01) using a hypergeometric test. B) Biological process enriched terms; C) Molecular function enriched terms and D) Cellular component enriched terms.

While pairwise analysis of transcriptional data collected from numerous parasite lifecycle stages generated a wealth of information related to schistosome developmental biology, a cohort of constitutively expressed gene products was also uncovered in our analyses. Here, gene product open reading frames (ORFs) that displayed the lowest 1% variation in expression across the schistosome lifecycle were interrogated for Gene Ontology (GO) term enrichment (Fig. 3 and Dataset S5). The majority of these constitutively expressed gene products were highly transcribed (Fig. 3A) and have GO term annotations that place their putative functional role in the fundamentally important ‘house-keeping’ processes of metazoan life (Fig. 3B–D). *S. mansoni* alpha tubulin (SCMSAT1A, Smp_103140), a gene product used extensively as a reference for quantitative reverse transcription PCR analyses [16],[30],[35],[36],[37],[38],[39], was found within this dataset. Forty-one constitutively expressed gene products specific to the phylum Platyhelminthes (as determined by the BLAST criteria within the *Methods*), and thus potential drug or vaccine candidates, were also identified in this analysis (Dataset S5).

### Network analysis of schistosome development

**Figure 4:**
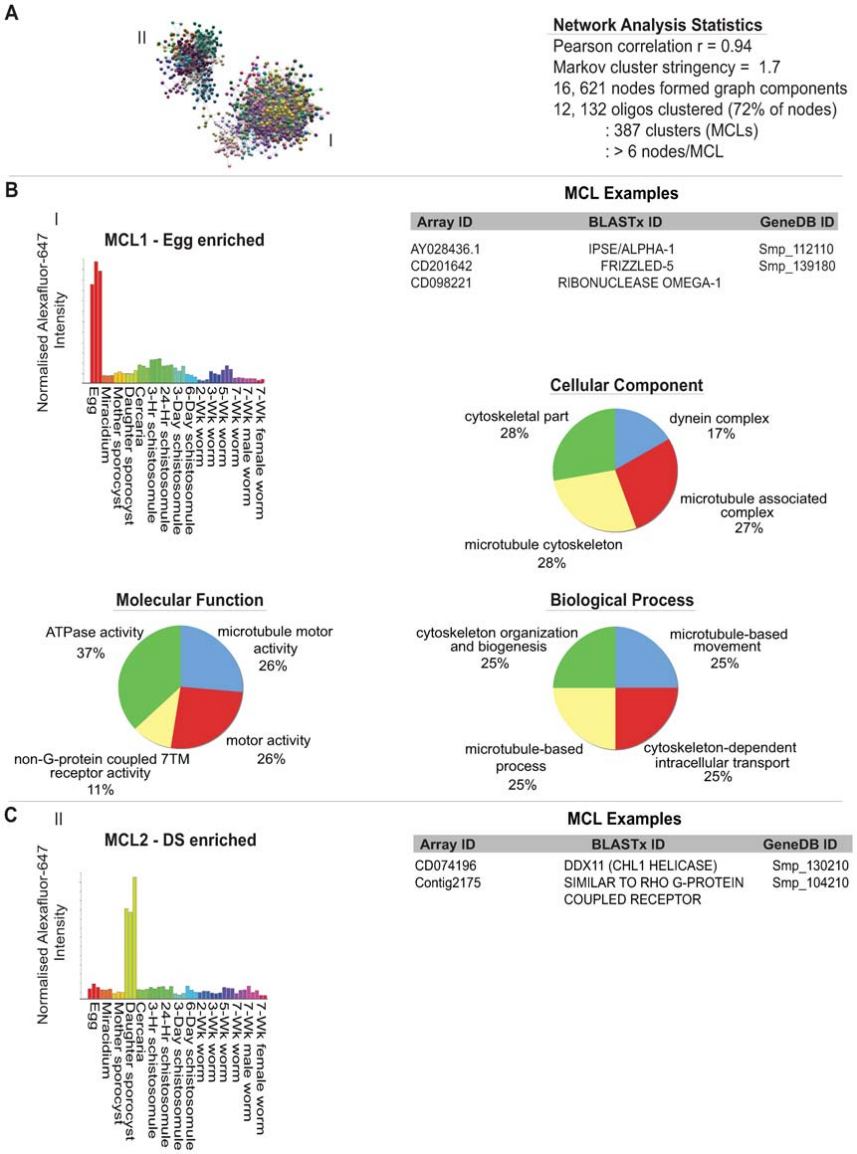
Network analysis of the schistosome lifecycle identifies egg and daughter-sporocyst enriched transcripts. Network analysis was performed as described in *Methods* using the BioLayout Express^3D^ package [29]. A) One network graph component containing two major clusters is illustrated (I; egg enriched transcripts and II; daughter sporocyst enriched transcripts). Different Markov clusters (MCL) within the network are represented by different coloured nodes (genes). The network analysis was performed using a Pearson correlation of r = 0.94 and Markov cluster stringency of 1.7 with accompanying details indicated. Nodes included in the identified 387 MCLs are included in Dataset S6, and form the basis of all subsequent descriptions. B) MCL1 includes transcripts highly enriched in the egg lifecycle stage (I). Some representative MCL gene examples are provided in addition to the GO terms significantly enriched in this cluster. C) MCL2 contains transcripts highly enriched in the daughter sporocyst lifecycle stage (II). Some representative MCL gene examples are provided. Bar graphs represent average normalised AF647 intensity values (Dataset S4) for all transcripts in that cluster, with replicate values obtained from independent life-stage hybridizations indicated as identical colours.

To complement the above-described pairwise analyses, we additionally performed a network investigation of gene expression across the schistosome's lifecycle. Briefly, network analysis (comprised of graphs created by nodes and edges) allowed the position of each schistosome transcript (nodes) to be placed within the context of all other differentially expressed transcripts (edges) throughout the parasite lifecycle. Pearson correlations between the normalised AF647 intensity values (Dataset S4) of every gene were generated and the data matrix (correlation threshold of r = 0.94) was used to interrogate the schistosome lifecycle. The stored data were used to draw the edges (lines) between nodes (transcripts) in network graphs. Nodes sharing edges (relationships above the set threshold) formed into identically coloured groups (graph components). Connectivity between nodes determined the 3-dimensional layout of the graphs. Using a Pearson correlation cut off of r = 0.94, 16,621 nodes (containing 12,132 oligonucleotides) formed graph components. One particular graph component containing two clusters of co-regulated genes (I: egg and II: Daughter sporocyst lifecycle stages) is illustrated (Fig. 4A).

**Figure 5 pntd-0000543-g005:**
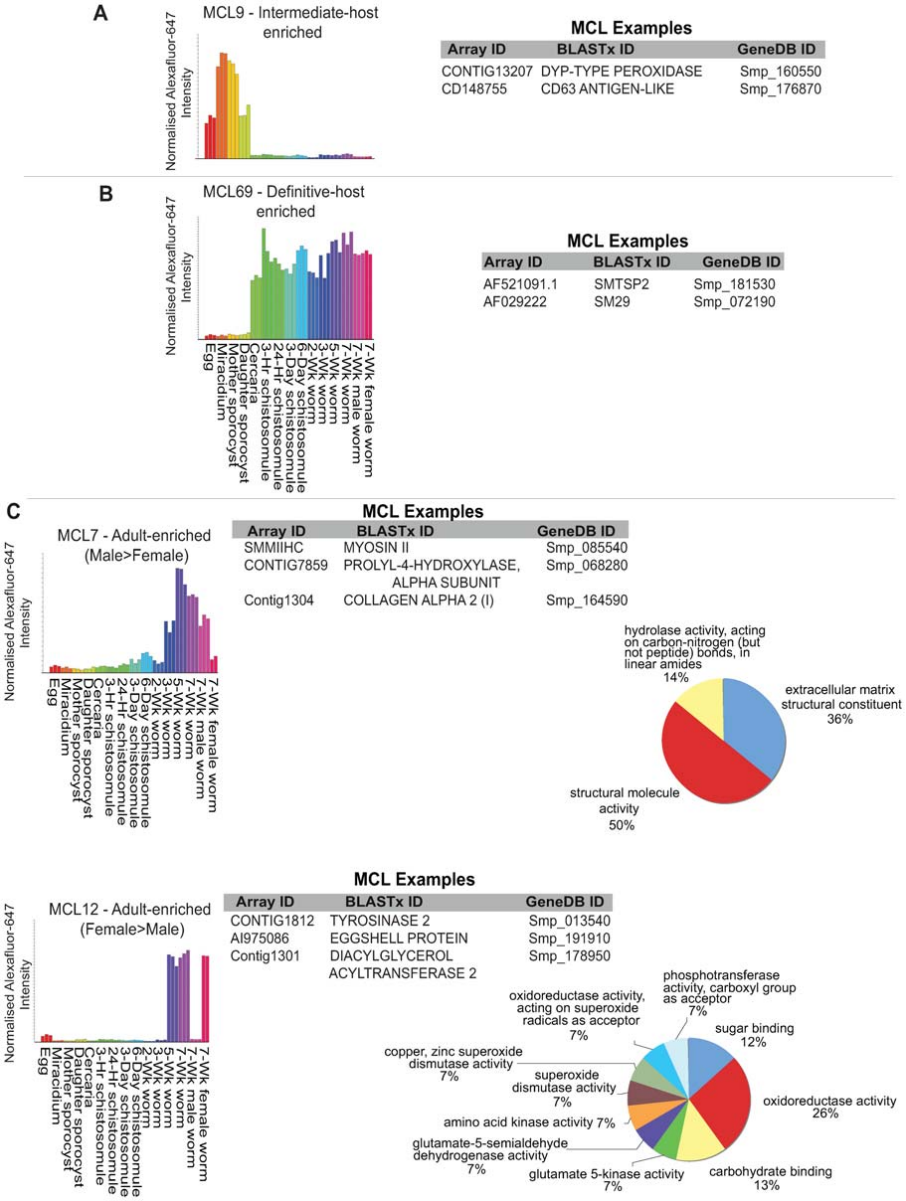
Markov clustering expands the repertoire of differentially expressed transcripts associated with the schistosome dioecious and digenetic state. Network analysis was performed as described in *Methods* (Pearson correlation, r = 0.94 and Markov cluster stringency  = 1.7). Selected MCLs are included to illustrate transcripts enriched in the intermediate host (A, MCL9), definitive host (B, MCL69) or in adult male and female schistosomes (C, MCL7 - Male > Female; MCL12 - Female > Male). In each case, specific examples of transcripts included in the selected MCLs are listed (full list of transcripts included in each cluster is found in Dataset S6). Enriched Gene Ontology Molecular Function categories are also included where identified. Bar graphs represent average normalised AF647 intensity values (Dataset S4) for all transcripts in that cluster, with replicate values obtained from independent life-stage hybridizations indicated as identical colours.

**Figure 6 pntd-0000543-g006:**
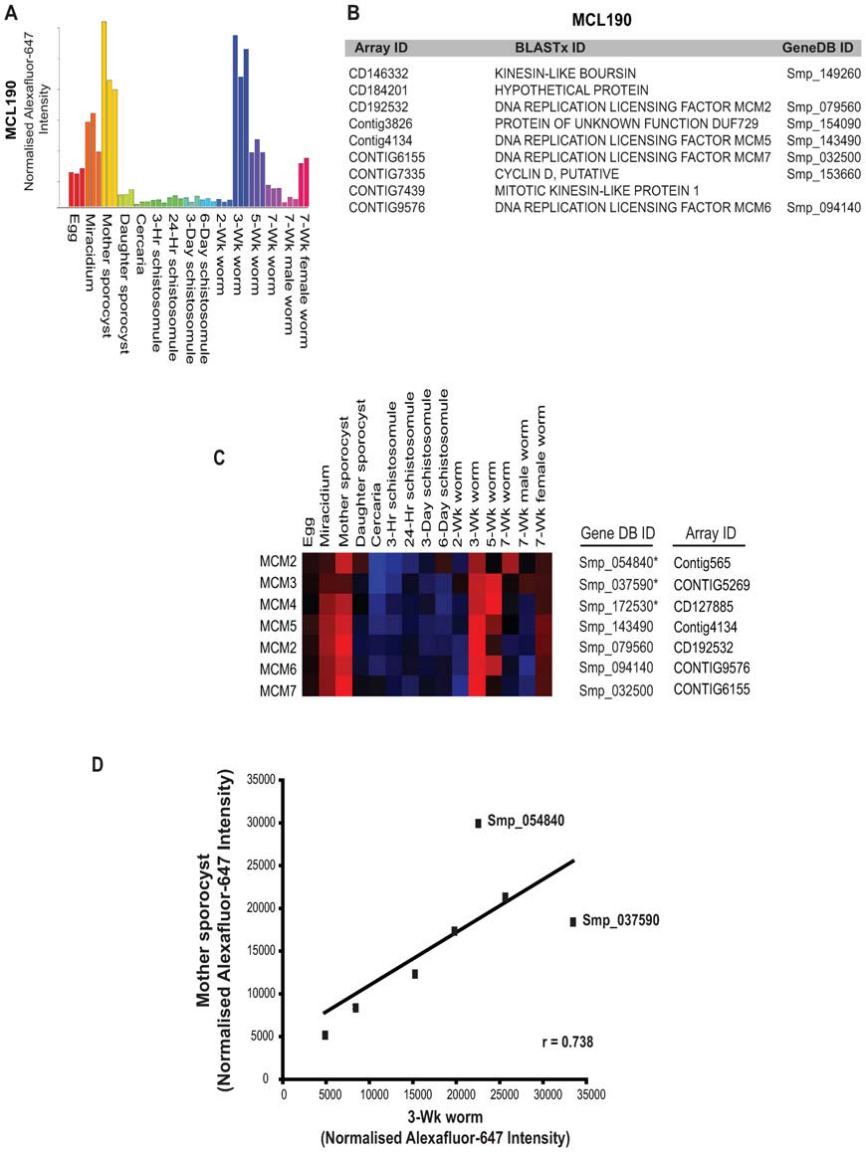
The minichromosome maintenance (MCM) heterohexamer is coordinately expressed in both mother sporocysts and 3-week adult worms. A) MCL190 identified 9 gene products sharing a peak of gene expression in the mother sporocyst and 3-wk adult worms. Bar graphs represent average normalised AF647 intensity values (Dataset S4) for all transcripts in MCL190, with replicate values obtained from independent life-stage hybridizations indicated as identical colours. B) List of genes contained within MCL190 demonstrates the presence of 4 out of the 6 transcripts constituting the eukaryotic MCM heterohexamer. C) Heatmap (Spearman's rank correlation coefficient, average linkage clustering, red – highly expressed, blue – weakly expressed) of all 6 MCM heterohexamer subunits (Dataset S10, including two MCM2 paralogs) defined by both array and GeneDB identifiers (ID). * indicates three MCM transcripts not clustered within MCL190. D) Scatterplot of MCM heterohexamer normalised AF647 intensity units in mother sporocyst versus 3-wk worm samples. A correlation coefficient of 0.738 suggests coordinated expression of the MCM heterohexamer with individual outliers (Smp_054840 and Smp_037590) indicated.

Large graphs were further subdivided into groups, or clusters of nodes, using a Markov Cluster algorithm (MCL). MCL clusters included transcripts that were statistically differentially expressed across groups (i.e. Adj*p*-value <0.05) as well as those that, although exhibiting similar profiles, were not statistically significant (examples in Figs. 5 and 6). This type of analysis, therefore, allowed for statistical associations to be verified, and was capable of identifying associations which statistical analyses (on minimally replicated datasets) could not detect. MCL clustering applied to this dataset at a stringency of 1.7 identified 387 clusters (MCL1-387) of 6 or more nodes displaying differential gene expression across the schistosome lifecycle (i.e. 12,132 nodes or 73% of the original dataset, Fig. 4A and Dataset S6).

Within the representative graph component (Fig. 4A), MCL analysis identified two major clusters that were highly enriched for egg and daughter sporocyst transcripts (MCL1 and MCL2, Fig. 4B and 4C, respectively). Within the egg-enriched dataset, 3,522 gene products were identified, of which IPSE/alpha-1 (AY028436.1, Smp_112110), fizzled-5 (CD201642, Smp_139180) and omega-1 (CD098221, no corresponding Smp) were members. As both IPSE/alpha-1 and omega-1 were previously identified as being expressed only in the egg stage of schistosome development [40],[41], the remaining gene products contained within MCL1 are likely to shed additional light on the biology of this immunopathogenic lifecycle stage. Gene ontology analysis of egg-associated transcripts contained within MCL1 (Fig. 4B) additionally suggested that processes involving microtubules and the cytoskeleton were highly enriched here. Investigation of the transcripts enriched within the daughter sporocyst stage (Fig. 4C and Figure S6) uncovered 1,509 gene products associated with this particular intramolluscan, schistosome developmental form. Interestingly, 55% (834/1,509) of these daughter sporocyst enriched transcripts had no known sequence similarity in NCBI databases (currently annotated as unknown or hypothetical proteins). Within sequences that have database hits, a rhodopsin-like GPCR (SmRHO [42]: Contig2175, Smp_104210) and a DNA helicase (DDX11 ortholog: CD074196, Smp_130210) were identified. As very few of these daughter-sporocyst enriched transcripts could be mapped to SchistoGeneDB (via their 50-mer long oligonucleotide representations, see *Methods*), no corresponding Gene Ontology classifications (similar to that performed for the egg enriched transcripts) could be identified.

Examination of the remaining 385 MCLs revealed clear examples of stage-specific gene expression, with network analysis being very good at teasing apart developmentally-regulated transcription in all three environmental niches occupied by the schistosome (intermediate host, vertebrate host and freshwater). While it is impossible to illustrate all of these clusters here (full list included in Dataset S6), a few examples (Figs. 5 & 6) will be described. Gene products contained within MCL9 represented those schistosome transcripts preferentially expressed in the lifecycle stages interacting with the intermediate host (Fig. 5A). While 122 of the 141 transcripts identified here were also found using pairwise statistical comparisons (Adj*p* <0.05, Table 1, Fig. 2A and Datasets S2 & S3), 19 gene products were newly discovered. Interestingly, one of these products (Dyp-type peroxidase: CONTIG13207, Smp_160550) is a member of a recently reviewed atypical heme peroxidase family [43] that appears to be expanded within the Platyhelminthes and not present within vertebrates [8].

MCL69 contains schistosome transcripts (Fig. 5B) predominantly expressed in life-stages interacting with the definitive host (as do MCL42, MCL210 and MCL259, see Dataset S4). The majority of these 18 transcripts (17/18) were also identified by pairwise analyses (Adj*p* <0.05, Table 1, Fig. 2A and Datasets S2 & S3), providing a clear example of how two different bioinformatics approaches led to the identification of identical stage-associated information (with SmTSP2 [18]: AF521091.1, Smp_181530 and Sm29 [44]: AF029222, Smp_072190 representing examples). Analysis of transcripts clustered within MCL7 and MCL12, however, revealed the full capability of network analysis by identifying additional genes associated with the adult dioecious state (Fig. 5C). Here, 129 further transcripts were identified as being male-associated in the adult (195 total with 66 previously identified by pairwise comparisons, Adj*p* <0.05, Table 1, Fig. 2A and Datasets S2 & S3) and 47 new transcripts found to be predominantly expressed in the adult female schistosome (93 total with 46 previously identified by pairwise comparisons, Adj*p* <0.05, Table 1, Fig. 2A and Datasets S2 & S3). Examples of specific network identified transcripts within these clusters are illustrated (Male - myosin II: SmMIIHC, Smp_085540; prolyl-4-hydroxylase alpha subunit: CONTIG7859, Smp_068280; collagen alpha 2 (I): Contig1304, Smp_164590 and Female – tyrosinase 2: CONTIG1812, Smp_013540; eggshell protein: AI975086, Smp_191910; diacylglycerol acyltransferase 2: Contig1301, Smp_178950)). Corresponding GO enriched Molecular Function categories within the adult male (MCL7) and female (MCL12) clusters confirmed well-documented functional differences [38] between the adult sexes. Here, adult male schistosomes displayed an enrichment of transcripts involved in structural biology whereas adult female parasites contain increased transcript representation within oxidoreductase, glutamate-5-semialdehyde dehydrogenase, glutamate 5-kinase and phosphotransferase activities (Fig. 5C).

While the network analysis was capable of detecting additional transcripts displaying developmental expression (when compared to the datasets generated by pairwise comparisons, Datasets S2 & S3), this approach was also able to identify new clusters of developmentally regulated transcripts that were not detected by statistical analyses. One example is MCL190 (full list can be found in Dataset S6), which included two peaks of expression; one in the asexually proliferating, mother sporocyst stage and one in the sexually maturing, 3-wk adult schistosome stage (Fig. 6A). Interesting, all transcripts within this cluster are involved in eukaryote DNA replication processes (except the hypothetical protein, CD184201, no Smp, Fig. 6B and Figure S10) with four of the six essential DNA helicase subunits ([45] MCM, minichromosome maintenance subunits 2, 5, 6 and 7) included. Searching SchistoGeneDB [25] for the remaining two components of the core MCM heterohexamer (MCM3, Smp_037590, CONTIG5269; MCM4, Smp_172530, CD127885) also revealed a second MCM2 gene prediction (Smp_054840, Contig565, possibly suggestive of an ‘unusual MCM’ as previously described [45]). While these schistosome MCMs were found outside MCL190, a heatmap representation of all MCM transcripts revealed the co-expression of the heterohexamer in both mother sporocyst and 3-wk parasite lifecycle stages (Fig. 6C), which was highly correlated (r = 0.738, Fig. 6D).

### Identification of developmentally regulated, Platyhelminthes-specific, schistosome transcripts

Schistosome genes expressed predominantly in the invading cercariae, tissue migrating schistosomula and/or blood residing worms are likely to participate in host immunomodulation, host immunoevasion and establishment of long-term, copulating, dioecious partners. We were interested in identifying those gene products, expressed in these particular lifecycle stages, which had no significant sequence similarity (see *Methods* for criteria) to NCBI database entries outside the phylum Platyhelminthes. These products would, therefore, represent another pool of attractive targets for anti-schistosome intervention strategies. We selected 37 MCLs (7, 35, 37, 38, 42, 45, 46, 65, 69, 77, 79, 94, 135, 171, 177, 183, 185, 187, 210, 214, 221, 222, 223, 259, 285, 287, 299, 301, 304, 319, 326, 336, 337, 338, 340, 348 and 350) as the 612 transcripts included herein displayed the queried transcriptional profile (expression in cercariae to schistosomula transformation and/or continued expression into the adult stage). A combined series of BLAST queries led to the identification of 294 gene products that only contained significant sequence similarities to schistosome or other Platyhelminth species. A heatmap representation (and oligonucleotide ID) of these 294 transcripts' normalised mean fluorescent intensities is depicted in Dataset S6.

### Global expression profiling of specific schistosome gene families

**Figure 7:**
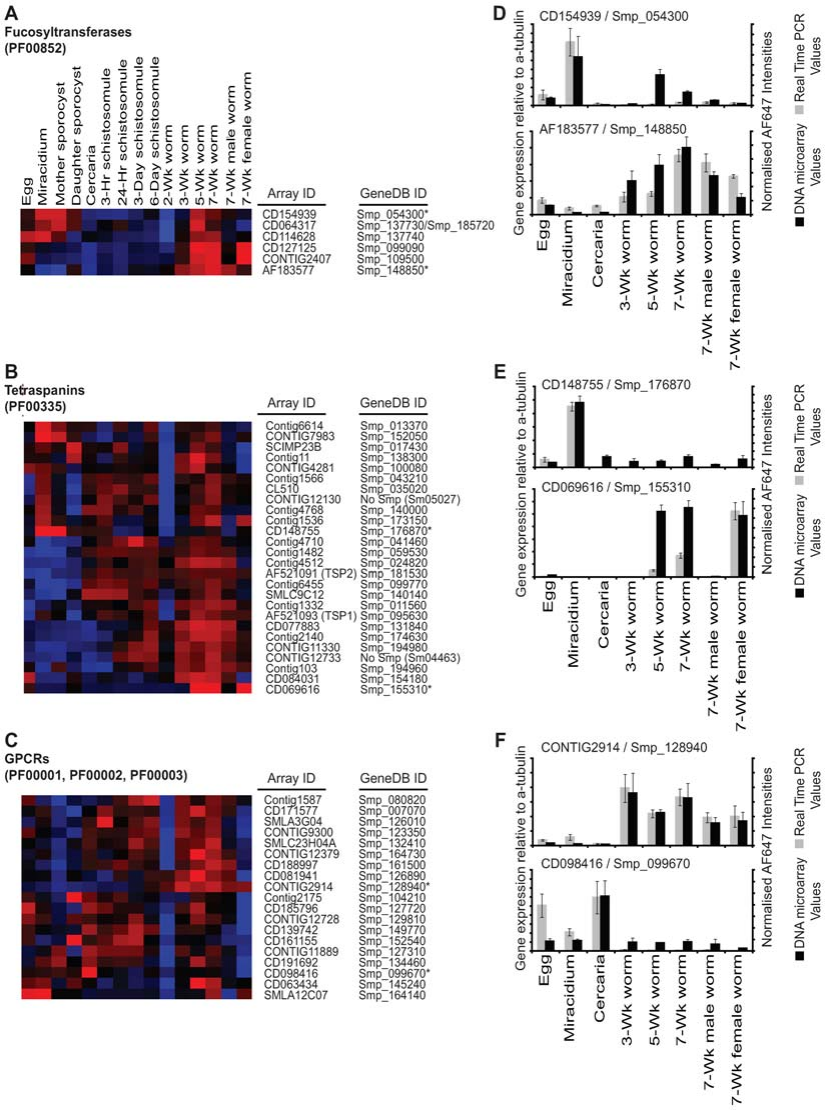
Analysis of three important schistosome gene families reveals differential transcription of individual members across the parasite's lifecycle. Using the latest version of the schistosome genome assembly (version 4.0), all putative fucosyltransferase- (PF00852, Dataset S7), tetraspanin- (PF00335, Dataset S8) and GPCR- (PF00001, PF00002 and PF00003, Dataset S9) family members were identified. DNA microarray oligonucleotides were mapped to specific regions of the predicted genes using the *S. mansoni* genome browser function of *S. mansoni* GeneDB [25]. For those predicted gene family members where 50-mer oligonucleotides could be mapped, expression data was extracted and analysed. Heatmap representation of all *S. mansoni*: A) fucosyltransferases (PF00852), B) tetraspanins (PF00335) and C) GPCRs (PF00001, PF00002 and PF00003) differentially expressed across the parasite's lifecycle (all data included in Datasets S7–9. Heatmaps (red – highly expressed, blue – weakly expressed) were created from normalised AF647 intensities using a Spearman's rank correlation coefficient and average linkage clustering (*Methods*). Corresponding real time RT-PCR confirmation of selected family members (*) (D – fucosyltransferases: CD154939/Smp_054300 & AF183577/Smp_148850; E – tetraspanins: CD148755/Smp_176870 & CD069616/Smp_155310; F – GPCRs: CONTIG2914/Smp_128940 & CD098416/Smp_099670) is indicated to the right of heatmap.

This DNA microarray characterisation of the schistosome lifecycle, as described by two different data analysis methodologies, provided a rich resource for novel biological discoveries. However, global profiling of the schistosome lifecycle additionally allowed for transcriptional comparisons of individual members within specific gene families to be conducted. Transcriptional ‘snapshots’ can provide important evidence for the selection/rejection of gene family members in the context of novel vaccine candidate or drug target identification and characterisation, saving valuable time in the pipeline of schistosomiasis control initiatives. We focused our analysis on three major gene families: fucosyltransferases (enzymes responsible for producing many of the immunomodulatory structures during infection [20]), tetraspanins (proteins that contain 4 transmembrane domains and are capable of inducing protective anti-schistosomal immunity [18]) and GPCRs (proteins that contain 7 transmembrane domains and are the most common targets of all clinically prescribed drugs [19]). We used PFAM [46] searches of SchistoGeneDB [25] to identify all predicted schistosome gene products within each family (PF00852 – fucosyltransferases; PF00335 – tetraspanins; PF00001, PF00002 and PF00003 – GPCRs). Clustering of all representative intra-gene family members provided a non-redundant list of schistosome fucosyltransferases (Dataset S7), tetraspanins (Dataset S8) and GPCRs (Dataset S9) to facilitate individual gene expression comparisons across the lifecycle. It is clear from the generated heatmaps of normalised mean fluorescent intensities that quite distinct gene expression profiles were identified within each gene family (Fig. 7). Expression profiles associated with the fucosyltransferase gene family were split between those members transcribed only in post-schistosomula stages compared to those members expressed in both snail-residing and mouse-residing parasite lifecycle stages (Fig. 7A). In contrast, the tetraspanins (Fig. 7B) and GPCRs (Fig. 7C) collectively displayed more diverse transcriptional patterns, possibly indicating complex stage-specific roles associated with individual family members. Real time quantitative PCR was used to verify the expression of several fucosyltransferase (Fig. 7D), tetraspanin (Fig. 7E) and GPCR (Fig. 7F) family members across the schistosome lifecycle.

## Discussion

Successful interrogation of large transcriptomic datasets depends upon appropriate analysis and bioinformatics tools to facilitate the effective bridging of phenotypic discovery to functional application. Within the field of schistosomiasis, large transcriptomic databases are being routinely created due to the current availability and use of several long-oligonucleotide DNA microarrays [13],[14],[16],[38]. This trend will continue as soon as high-throughput sequencing platforms become financially more accessible to academics and the use of these platforms is no longer restricted by the bottleneck of current computational sequence assembly algorithms. However, all high-throughput technologies require the most effective strategies for elucidating biologically meaningful information from complex transcriptome datasets. Towards this end, we describe a combinatorial bioinformatics strategy for analysing transcriptome data collected from the hybridisation of 15 different schistosome lifecycle stages, spanning three unique environmental niches and two distinct hosts, to a long-oligonucleotide DNA microarray consisting of 37,632 elements.

Initial analyses of normalised fluorescent DNA microarray data collected from the schistosome lifecycle immediately illustrated a high degree of correlation, in all but two cases, across the 15 profiled developmental forms (Fig. 1). This suggested that, while clear stage and sex-specific transcriptional differences can be identified (Figs. 2 and 4–7), many schistosome transcriptional processes are coordinately regulated to a degree, independent of the environmental niche that the parasite occupies. Interestingly, transcripts from both the short-lived cercariae and miracidia lifecycle stages had highly correlated, normalised fluorescent intensities when compared to those collected from any of the long-lived adult parasite life stages (e.g. 2-wk, 3-wk, 5-wk or 7-wk mixed-sex schistosomes). This indicated that the infective lifecycle stages are not as transcriptionally silent as previously perceived [4] or, equally possible, that they are able to stably store a large repertoire of transcripts important for infection and lifecycle progression [47]. Additionally, this correlation analysis demonstrated that the transcriptional profiles collected from egg and daughter sporocyst lifecycle stages were outliers when compared to other schistosome developmental forms. Classical studies of the schistosome egg have clearly indicated that this lifecycle stage is involved in complex biological phenomena including the induction of host immunopathologies [48], and more important to our results presented here, production of snail-infective miracidia. As miracidia development within the egg progresses through five morphologically distinguishable stages (Stages 1–5, as described by Vogel [49]) and the egg material used in this study included all five, it is likely that lack of correlation to other developmental forms was directly linked to the biological intricacy of egg maturation. Transcriptional profiling of fractionated egg samples [50] will likely shed more light on the intricate biology of egg maturation in future investigations. Similarly, the complex biology associated with the daughter sporocyst, involving both sporocystogenesis and cercariogenesis [51], provides a rational explanation for the lack of correlation observed between this developmental form and others within the lifecycle. Therefore, simple correlation analyses of normalised gene expression data collected across the schistosome lifecycle provided an immediate insight into parasite developmental biology and identified the two most transcriptionally distinct developmental forms.

More detailed approaches, involving both pairwise and network based transcriptomic analyses, were next used to interrogate parasite lifecycle progression. Although pairwise analyses detected stage and sex-associated gene products of statistical significance (Table 1, Dataset S2), the inherent experimental background found in large-scale microarray datasets, collected from minimally replicated experiments (due to difficulties in obtaining schistosome material), forced the use of multiple testing corrections to limit false positive identification. While this restricted our analysis to 973 gene products differentially expressed in at least one pairwise comparison (Fig. 2A), the dataset was deemed to be a robust and strictly-selected representation of schistosome developmental biology. It is likely that the use of alternative statistical methods for transcriptome analysis (e.g. ANOVA) would generate complementary datasets of schistosome development. This was beyond the scope of the current study, but could be revisited using primary data deposited within EBI ArrayExpress [28].

The ease by which statistical methods identified the most transcriptionally divergent points in the schistosome lifecycle, namely those related to the dioecious and digenetic state (Fig. 2A and Dataset S3), was readily apparent. While many of these developmental and sex-associated transcriptional profiles supported previous observations (e.g. cathepsin B, Contig1226 [52] and superoxide dismutase, SCMSOD1 [37],[38],[53]), we uncovered a variety of additional transcripts (e.g. netrin: CD167333, Smp_146840; the netrin receptor: CD168439, Smp_165440; leishmanolysin: CONTIG7854, Smp_173070; CD164149, Smp_173070; CONTIG7744, Smp_173070; and CD133033) that displayed differential expression across the schistosome lifecycle (full list in Dataset S3). Effectively, these transcripts (particularly those also displaying >32 fold differential expression, Fig. 2B and Dataset S3) represent the highest quality dataset, to date, that accurately describes parasite transcriptional patterns across three environmental niches. As performed recently for neuronal cell cultures [54], it would be interesting to identify the presence of conserved transcriptional regulatory features (i.e. amongst promoters or untranslated regions (UTRs) of coordinately expressed genes) within this developmentally-regulated dataset. While this was outside the scope of the present study, such information could lead to advances into the mechanisms underpinning gene expression regulation between the adult sexes or across developmental stages of this platyhelminth.

The co-regulation of both netrin and netrin receptor during sexual maturation in the definitive host (peaking in 5 wk and 3 wk adult worms, respectively) is noteworthy as this receptor/ligand interaction is absolutely essential for somatic and gonadal development in *Caenorhabditis elegans* [55] as well as central nervous system (CNS) regeneration in *Schmidtea mediterranea* [56]. Inhibiting netrin/netrin ligand interaction may negatively influence schistosome sexual maturation and CNS development, thus providing an opportunity for intervention. The inverse developmental regulation of three putative, cell-surface leishmanolysin-like proteases (two intermediate host-enriched isoforms: CONTIG7854, CD164149 and CONTIG7744 and one definitive-host enriched isoform: CD133033) is also notable due to their proposed functional roles in parasite invasion mechanisms and immune evasion strategies [57]. During leishmaniasis, leishmanolysin-like proteins are involved in inactivation of complement C3 [58], inhibition of host cell interactions [59], perturbations in host cell signaling [60] and degradation of the extracellular matrix [61]. It is tempting to speculate that the schistosome leishmanolysin-like orthologs impart analogous activities during host/parasite interactions within snails and vertebrates, collectively favouring the development of schistosomiasis. Indeed, secreted/excreted leishmanolysin-like proteins have recently been reported during cercariae/schistosomula [62] and miracidium/mother sporocyst [63] transformation and provides evidence for this hypothesis. Inverse lifecycle expression of duplicated gene family members has been observed for the SmVALs and probably represents the schistosome's evolutionary solution for niche specialization as has previously been described [30]. As these leishmanolysin-like proteases represent another expanded gene family (along with the Dyp-type peroxidases, Fig. 5A) within the *Schistosoma* (orthologs not present in vertebrate genomes), targeting members expressed in key life-stages could lead to the selective development of novel intervention strategies.

Statistical analyses also made it straightforward to identify those schistosome gene products that were constitutively expressed across the parasite's lifecycle. Here, we examined the lifecycle dataset for transcripts that did not display differential expression in any pairwise comparison and further selected only those individuals that exhibited invariant (less than 1% variation) normalised fluorescent intensities in each lifecycle stage (Fig. 3). This analysis identified 338 ‘housekeeping’ gene products (709 with less than 2% variation, data not shown), which expands the number of schistosome transcripts considered to be absolutely essential for lifecycle progression. While many of these transcripts were linked to conserved biological activities essential for metazoan life (Fig. 3B–D), 41 gene products displayed no sequence similarity outside of the Platyhelminthes (Dataset S5) and, thus could be considered as a pool of novel anthelmintic targets. This approach in drug target identification is in direct contrast to that proposed by Caffrey *et al.* [64]. However, it is likely that both methodologies will lead to significant progress in therapeutic discovery and therefore, further characterisation of these Platyhelminthes-associated, constitutively-expressed, schistosome gene products is ongoing.

Since traditional pairwise comparisons of DNA microarray datasets do not take into account the higher order structure of the compared entities, it was impossible to determine how each schistosome transcript interacted with its neighbours using this statistical methodology. As network analysis examines large-scale DNA microarray datasets quite differently [17],[65],[66],[67],[68], we applied this complementary approach in transcriptome analysis to the schistosome lifecycle. This permitted formation of higher order structures within the dataset, effectively considering how each schistosome transcript interacted with all other neighbouring transcripts across the 15 lifecycle stages. Smaller distances of edges between nodes represented highly correlated gene expression, whereas larger distances of edges between nodes represented poorly correlated gene expression (e.g. Fig 4A). Markov Clustering algorithms (MCLs, [17],[69]) subsequently were used to subdivide large network graphs into clusters of co-regulated schistosome genes (Figs. 4B–D, Fig. 5, Fig. 6 and Dataset S6).

While the parameters used for the network analysis were strict (Pearson correlation, r = 0.94, Fig. 4A), 16,621 oligonucleotides still passed these criteria. MCL analysis clustered 12,132 of these oligonucleotides (72%) into 387 groupings (with at least 6 oligonucleotides/MCL cluster, Dataset S6) and this dataset was subjected to further investigations. Many patterns of differential gene expression (containing genes previously identified by the statistical-based approaches used herein) were confirmed by network analysis (intermediate-host enriched, Fig. 5A; definitive-host enriched, Fig. 5B and adult sex-associated, Fig. 5C). Revealingly, within these clusters were additional genes, harbouring identical patterns of co-expression, which were not detected by the statistical-based approaches (full list in Dataset S6). Furthermore, network-based techniques facilitated the discovery of key life-stage associated transcripts (e.g. egg-associated, Fig. 4B; daughter sporocyst-associated, Fig. 4C), which were not revealed by statistical-led approaches leading to a more complete view of schistosome developmental biology. As an example, the biphasic up-regulation of four out of the six core, eukaryote DNA helicase subunits (MCM2, 5, 6 and 7) in mother sporocysts and 3-wk worms clearly indicated that these two lifecycle stages are more heavily involved in DNA replication processes than any other examined schistosome developmental form (MCL190, Fig. 6A–B). Interestingly, MCMs have been implicated in other activities including transcriptional regulation, chromatin remodeling and checkpoint responses [45]. Mother sporocysts are actively involved in asexual reproduction of the germinal cell-containing daughter sporocysts within the intermediate host and 3-wk worms are beginning to develop the gamete-producing organs (ovaries and testes) required for sexual reproduction within the definitive host. Therefore, this transcriptional finding has a biological rationale and may point to these two life-stages as being critical checkpoints in schistosome lifecycle progression. The important point here is that network exploratory processes, and not standard statistical methods, identified these MCMs (and MCM3 and 4, Fig. 6C–D and Dataset S10) as being co-regulated.

Further interrogation of the network data led to the identification of 37 MCLs predominantly expressed in the definitive host interacting cercariae and/or schistosomula and/or adult developmental stages (612 transcripts contained within MCLs 7, 35, 37, 38, 42, 45, 46, 65, 69, 77, 79, 94, 135, 171, 177, 183, 185, 187, 210, 214, 221, 222, 223, 259, 285, 287, 299, 301, 304, 319, 326, 336, 337, 338, 340, 348 and 350). Included in these MCLs were many schistosome gene products that display strong sequence similarity to NCBI database entries collected from a wide range of taxonomic groups. While these specific, annotated products were of general interest in terms of developmental biology, there were 294 schistosome transcripts, which shared little or no sequence similarity to NCBI entries outside of the phylum Platyhelminthes (Dataset S6). These specific transcripts may complement those similarly identified constitutively expressed schistosome gene products (Fig. 3 and Dataset S5) and collectively be considered paramount in the search for novel anti-schistosomal therapeutic targets. Indeed, this genome exclusion approach is currently being used for the identification of novel anti-fungal and anti-trypanosomatid drug targets [70],[71].

One final use of this large transcriptome dataset was to comparatively profile individual members of important gene families proposed as vaccine candidates, drug targets and immunomodulatory enzymes. Firstly, this analysis (Datasets S7–S9 and Fig. 7) implicates several new tetraspanins (PF00335, Tsps) as vaccine candidates, based on the successful immunoprophylactic results previously reported for the surface-exposed, tegumental antigens SmTsp1 and SmTsp2 [18]. As both SmTsp1 and SmTsp2 are predominantly expressed during cercariae to adult worm development (Fig. 7B), other tetraspanins displaying this transcriptional profile could be considered as novel immunoprophylactic targets. Based on the DNA microarray data (Fig. 7B and Dataset S8), many tetraspanins meet this criterion including CONTIG12733 (Sm04463) and Contig4768 (Smp_140000). Interestingly, these two tetraspanins were also found localised on the outer tegument (surface) of *S. mansoni* adults in a recent proteomic investigation [72]. As the tegument is generally viewed the most susceptible structure to host-mediated immune attack [73], Sm04463 and Smp_140000 should be considered as priority antigens in the search for novel vaccine candidates. The localisation of the remaining tetraspanins will help guide a rational strategy for future immunoprophylactic investigations of this family.

Secondly, this analysis identified several schistosome G-protein coupled receptors (GPCRs) as potential new drug targets (Fig. 7C). GPCRs are 7-transmembrane domain spanning proteins constituting the largest and most divergent class of cell surface receptor and are among the most important of all drug targets: ∼30% of all clinically prescribed drugs function as GPCR agonists or antagonists [74]. GPCRs are therefore model drug targets and arguably amenable to novel medical and biotechnology applications. The latest *S. mansoni* genome assembly suggests that there are at least 65 non-redundant, PFAM indicated, GPCR sequences (likely an under-representation, see [9]) contained within the species (using PF00001, PF00002 and PF00003 criteria, Dataset S9), including the molecularly characterised histamine-responsive GPCR [75], the rhodopsin-like GPCR [42] and a dopamine-reactive GPCR [76]. Unfortunately, the long-oligonucleotide DNA microarray only contained unambiguous representations for a fraction of these GPCRs (19/65 or 30%). As GPCRs are generally expressed at much lower levels than most other metazoan genes [77], their under-representation on our long-oligonucleotide DNA microarray, developed predominantly from EST sequence information collected up until May 2005 [16], was therefore, not surprising. However, several examples of schistosome GPCRs with drug-target potential were found, including representative members expressed during schistosomula development (e.g. CD139742, Smp_149770; CD161155, Smp_152540 and CONTIG11889, Smp_127310) as well as those predominantly expressed during adult worm maturation (e.g. CONTIG2914, Smp_128940; CD081941, Smp_126890 and CD188997, Smp_161500). GPCR ligand identification could lead to the characterisation of anti-schistosome chemotherapeutic agonists/antagonists and is currently being pursued using the application of automated robotics [78].

Finally, this approach was used to identify the transcriptional profile associated with the schistosome fucosyltransferase (FucT) repertoire (PF00852, Fig. 7A). This important protein family enzymatically produces fucosylated glycolipids and glycoproteins, which in turn are responsible for type-2 immune response polarisation [79], circumoval granuloma formation [80] and egg/endothelium interactions [81] during schistosomiasis. Primary nucleotide similarity amongst schistosome family members is high (14 Smp locations assembled into 8 clusters, Dataset S7) and, therefore, only six oligonucleotides were useful for discriminating individual FucT lifecycle transcriptional patterns. Interestingly, FucT expression is divided into two major clades; those that are expressed predominantly in miracidia/mother sporocyst stages and those primarily expressed in sexually mature adults. Expression of FucT subsets within the intermediate snail host (CD154939, Smp_054300; CD064317, Smp_137730/Smp_185720 and CD114628, Smp_137740) may well be responsible for the distinctive fucosylation patterns exhibited by larval glycoproteins, which are thought to function in immune recognition by the snail's defense system [82]. Conversely, FucTs expressed in adult females and/or eggs (CD114628, Smp_137740; CONTIG2407, Smp_109500 and CD127125, Smp099090) may be responsible for the increased enzymatic activity detected in egg extracts [20] as well as the major biological processes (type-2 response induction, circumoval granulomatous inflammation and trans-endothelial migration) associated with schistosome eggs. If so, then inhibiting (i.e. via iminosugars) these specific FucTs may be a rational approach in preventing the formation of egg-mediated pathological immune reactions [48] and, therefore, these enzymes should be investigated as novel anti-schistosomal targets.

This study, combining network and statistical based analysis of the *S. mansoni* lifecycle, has thoroughly explored many of the transcriptional subtleties associated with developmental and sexual maturation of this pathogenic trematode. As a novel approach in discovery-led transcriptomics, these methods enabled a systems biology view to be attained, with many possible immunoprophylactic and therapeutic lead targets identified. It is envisioned that some of the candidates identified herein will be subjected to future hypothesis-led functional investigations. The completion of such specific examinations will ultimately contribute to the successful development of novel control strategies useful in the alleviation of schistosome-induced immunopathologies, morbidities and mortalities.

## Supporting Information

Dataset S1 Clustered sequences used for oligonucleotide design. Complete nucleotide sequences of the clustered schistosome dataset are provided. Clustering details are described in [16]. 50-mer oligonucleotide representations of each sequence were generated as described in [16].
(23.28 MB TXT)

Dataset S2 Selected pairwise comparisons of schistosome development. Multiple pairwise comparisons were performed as described in the Methods. The column description worksheet provides details as to each column header. Subsequent worksheets identify transcripts passing the applied statistical criteria in the lifecycle stages being compared.
(13.80 MB XLS)

Dataset S3 Lifecycle expression profiles of statistically identified gene products from pairwise comparisons of key schistosome developmental stages. The column description worksheet provides details as to each column header. The remaining two worksheets identify the transcripts passing the indicated statistical criteria (‘adj*p*<0.05’ OR ‘adj*p*<0.05 and >32 FDE’ worksheets).
(1.78 MB XLS)

Dataset S4 Normalised AF647 fluorescent intensity values of each oligonucleotide representation on the DNA microarray across the schistosome lifecycle. Normalisation procedures are described in the Methods. Replicate lifecycle stages are colour coded. ‘Name’ column header represents the oligonucleotide identifier. ‘Block, column, row and ID’ represent physical location of each 50-mer oligonucleotide on the DNA microarray.
(33.74 MB XLS)

Dataset S5 Schistosome transcripts demonstrating constitutive expression. The column description worksheet provides details as to each column header. Subsequent worksheets identify the constitutively expressed transcripts (less than 1% variation in expression across all life-stages examined, ‘constit_1pc_copy.txt’ worksheet), provide details as to Gene ontology (GO) enrichment (‘constit_1pc_GO.xls’ worksheet) and list those that have sequence similarity restricted within the Platyhelminthes (‘Platyhelminth restricted’ worksheet).
(0.54 MB XLS)

Dataset S6 Identity of schistosome transcripts found by network analysis within the 387 MCLs. The column description worksheet provides details as to each of the column headers. Each subsequent worksheet identifies transcripts found in each of the 387 MCLs and also includes the average transcription profile (bar chart of replicate values) of each MCL across the lifecycle. ‘MCLGO’ worksheet provides details as to which MCLs have enriched GO classifications included. ‘Develop Platyhelminth specific’ worksheet lists the differentially-expressed transcripts, described in the Methods, that only contain sequence similarity within the Phylum Platyhelminthes. A heatmap of these identified transcripts is also included.
(28.62 MB XLS)

Dataset S7 Expression profiles for all annotated fucosyltransferases (FucTs, PF00852) contained in the *Schistosoma mansoni* GeneDB version 4 assembly. All FucTs nucleotide sequences were downloaded from the *Schistosoma mansoni* GeneDB version 4 assembly and clustered according to the Methods. Details of this assembly are included in the ‘FucT assembly’ worksheet. The ‘FucT’ worksheet provides the normalised expression profiles for each FucT, where a 50-mer oligonucleotide could be mapped to the genome assembly. The ‘FucT RTPCR’ worksheet provides the details of FucT expression verified by real time PCR analysis. The ‘FucT for heatmap’ worksheet provides the average FucT expression values used for construction of the lifecycle heatmap depicted in Figure 7. All subsequent worksheets provide individual FucT gene expression profiles for 50-mer oligonucleotides mapped to Smp gene predictions.
(0.14 MB XLS)

Dataset S8 Expression profiles for all annotated tetraspanins (tsps, PF00335) contained in the *Schistosoma mansoni* GeneDB version 4 assembly. All tsp nucleotide sequences were downloaded from the *Schistosoma mansoni* GeneDB version 4 assembly and clustered according to the Methods. Details of this assembly are included in the ‘tsp assembly’ worksheet. The ‘tetraspanins’ worksheet provides the normalised expression profiles for each tsp, where a 50-mer oligonucleotide could be mapped to the genome assembly. The ‘tsp RTPCR’ worksheet provides the details of tsp expression verified by real time PCR analysis. The ‘Tsps for heatmap’ worksheet provides the average tsp expression values used for construction of the lifecycle heatmap depicted in Figure 7. All subsequent worksheets provide individual tsp gene expression profiles for 50-mer oligonucleotides mapped to Smp gene predictions.
(0.80 MB XLS)

Dataset S9 Expression profiles for all annotated GPCRs (GPCRs, PF00001, PF00002 and PF00003) contained in the *Schistosoma mansoni* GeneDB version 4 assembly. All GPCR nucleotide sequences (separated for PF00001, PF00002 and PF00003) were downloaded from the *Schistosoma mansoni* GeneDB version 4 assembly and clustered according to the Methods. Details of this assembly are included in the ‘GPCR assembly’ worksheet. The ‘GPCRs’ worksheet provides the normalised expression profiles for each GPCR, where a 50-mer oligonucleotide could be mapped to the genome assembly. The ‘GPCRs RTPCR’ worksheet provides the details of GPCR expression verified by real time PCR analysis. The ‘GPCRs for heatmap’ worksheet provides the average GPCR expression values used for construction of the lifecycle heatmap depicted in Figure 7. All subsequent worksheets provide individual GPCR gene expression profiles for 50-mer oligonucleotides mapped to Smp gene predictions.
(0.39 MB XLS)

Dataset S10 Summary of expression data collected for the schistosome minichromosome maintenance (MCM) heterohexamer. Mapping of 50-mer oligonucleotide representations to genome assembly version 4 of *Schistosoma mansoni* GeneDB. Average MCM expression values used for construction of lifecycle heatmap in Figure 6 are also included (‘MCM for heatmap’ worksheet).
(0.06 MB XLS)
